# The mediating role of body surface area-adjusted basal metabolic rate: effects of low muscle mass and central obesity on cognitive impairment in Chinese patients with type 2 diabetes mellitus

**DOI:** 10.3389/fendo.2024.1513035

**Published:** 2025-01-24

**Authors:** Ya-Jie Zhai, Fang Li, Chen-Ying Lin, Fan Wu, Hui-Na Qiu, Jing-Bo Li, Jing-Na Lin

**Affiliations:** ^1^ School of Medicine, Nankai University, Tianjin, China; ^2^ Department of Endocrinology, Tianjin Union Medical Center, Nankai University Affiliated Hospital, Tianjin, China; ^3^ Tianjin Union Medical Center, Tianjin Medical University, Tianjin, China

**Keywords:** basal metabolic rate, body composition, middle-aged and older people, cognitive impairment, obesity, type 2 diabetes mellitus

## Abstract

**Background:**

This study investigates the relationship between basal metabolic rate (BMR), body composition, obesity indices, and cognitive impairment (CI) in middle-aged and older type 2 diabetes mellitus (T2DM) patients, assessing their potential role in CI screening.

**Methods:**

A cross-sectional study included 1243 T2DM patients over 45 years old. CI was assessed using the Montreal Cognitive Assessment. BMR and body composition indices were measured through bioelectrical impedance analysis. The associations and predictions related to CI were explored using multivariable-adjusted logistic regression, restricted cubic spline (RCS) models, and receiver operating characteristic (ROC) curve analyses. Mediation analysis explored the role of BMR adjusted by body surface area (BMR/BSA) in CI risk.

**Results:**

Patients with CI showed significantly lower BMR, BMR adjusted for height squared (BMR/Height²), BMR/BSA, appendicular skeletal muscle mass (ASM), and fat-free mass (FFM), alongside higher waist circumference (WC) and percentage of body fat. Logistic regression showed that participants in the fourth quartile of BMR, BMR/Height^2^, and BMR/BSA had approximately a 54% reduced risk of CI (odds ratio range 0.457 to 0.463). RCS analysis indicated a linear decrease in CI risk with increasing BMR metrics. ROC analysis indicated high predictive efficacy for CI with combined indicators, particularly BMR and FFM (area under the curve 0.645). Mediation analysis suggested that BMR/BSA played a significant mediating role in WC, ASM and FFM on CI risk, with a mediation proportion ranging from 45.73% to 50.87%.

**Conclusion:**

Low energy expenditure assessed by BMR/BSA is an independent risk factor for increased CI risk in middle-aged and elderly T2DM patients. Central obesity, low muscle mass, and low energy expenditure significantly elevate CI risk in this population.

## 1 Introduction

Type 2 diabetes mellitus (T2DM) is a chronic metabolic disease characterized by glucose and fat metabolism disorders. According to statistics, in 2021, there are approximately 540 million diabetic patients worldwide, about 95% of which are T2DM patients (1). China has about 150 million diabetic patients, which is the country with the largest diabetic population (1). Cognitive impairment (CI) encompasses disorders marked by difficulties in understanding, attention, calculation, visuospatial skills, language, memory, and executive function, arising from various neurological, psychological, and physiological factors (2). Diabetes can lead to significant brain changes, contributing to CI (3). A meta-analysis indicated that nearly 45% of T2DM patients experience CI, with poor glycemic control accelerating its progression to dementia (4, 5). Additionally, individuals with CI impose a substantial economic burden on families and society due to increased disability, reduced self-management capabilities, and shortened life expectancy (6, 7). The mechanism of diabetes leading to CI mainly involves the activation of polyol pathway, the increase of advanced glycation end products, the activation of protein kinase C and the change of hexosamine pathway, which together lead to the deterioration of neurotransmitter function (8, 9), and ultimately lead to CI.

The basal metabolic rate (BMR) refers to the energy consumed by the body to maintain basic life activities in the awake state (10). It is closely related to muscle mass and fat distribution (11) and is usually measured about 10 hours after the last meal, or in a controlled thermally neutral environment (12). Compared with non-diabetic patients, BMR of diabetic patients is often higher (13). This phenomenon may be influenced by various factors, including increased levels of carbohydrate oxidation, enhanced gluconeogenesis and hepatic glucose output, and heightened sympathetic nervous activity, which collectively lead to alterations in metabolic activities within the body, further promoting an increase in BMR (14, 15). However, the relationship between diabetes and BMR is intricate and nuanced. Research indicates that individuals with long-term diabetes often experience a reduction in BMR (16). This decline can be attributed to several factors, including decreased hepatic glucose production, reduced lipid oxidation, and prolonged muscle atrophy, all of which ultimately contribute to a lower BMR (16–18).

Low muscle mass and central obesity are widely recognized as important factors influencing CI (19, 20). However, studies examining the effects of central obesity and low muscle mass on CI show conflicting results. Some research indicates that a decrease in muscle mass is closely associated with accelerated cognitive decline and an increased risk of CI (21, 22). Additionally, central obesity measured by high waist circumference (WC) is considered a risk factor for CI and dementia (23, 24). Conversely, other studies have found no significant association between skeletal muscle mass and WC with CI (25–27). Notably, low muscle mass and central obesity are often accompanied by a decrease in BMR, which affects overall energy balance (28). Research conducted by Luke A. and Dulloo A.G. indicates that low muscle mass is generally associated with a decrease in BMR (28, 29). The reduction in muscle mass is closely linked to the progressive loss of myocytes and an increased rate of degenerative changes, which together contribute to the declining trend in BMR (30). Similarly, studies by Stiegler P. and Ashtary-Larky D. have found that central obesity, as measured by high waist circumference, is often accompanied by reduced fat oxidation and increased fat storage, further exacerbating the decline in BMR (28, 31). Furthermore, a lower BMR can lead to metabolic dysfunction in both the body and brain, impairing glucose metabolism and resulting in insufficient oxygen supply to the brain. This deficiency can adversely affect neurotransmitter synthesis and may ultimately contribute to cognitive impairment, potentially progressing to dementia (32–34). Under the background of low muscle mass and high WC, low BMR may significantly elevate the risk of diabetes-related CI, which may explain the inconsistency of the effects of low muscle mass and central obesity on CI. However, there is currently a lack of studies investigating the correlation between CI and BMR in diabetic populations, as well as the potential mediating role of BMR in the relationship between low muscle mass and high WC concerning CI.

This study aims to evaluate the relationship between obesity-related indicators, body composition, BMR, and their height- and body surface area (BSA)-adjusted indices (BMR/Height² and BMR/BSA) with CI in middle-aged and elderly patients with T2DM. Specifically, body composition encompasses fat-free mass (FFM), appendicular skeletal mass (ASM), visceral fat area (VFA), and percentage of body fat (PBF), while obesity indicators include WC and body mass index (BMI). Furthermore, this research will explore the potential mediating role of BMR/BSA in the relationship between central obesity (WC) and low muscle mass (ASM and FFM) on CI risk, so as to provide a reliable basis for the early prevention of CI in middle-aged and elderly patients with T2DM.

## 2 Methods

### 2.1 Study design and participants

Our study is a cross-sectional analysis involving T2DM patients aged 45 and older, who were first admitted to the Endocrinology Department of Tianjin Union Medical Center between July 2020 and January 2024. These admissions were primarily for routine diabetes management, complications related to diabetes, and other endocrine disorders.

### 2.2 Inclusion and exclusion criteria

The inclusion criteria were individuals diagnosed with diabetes according to the diagnostic criteria established by the World Health Organization in 1999, aged ≥45 years.

**Figure 1:**
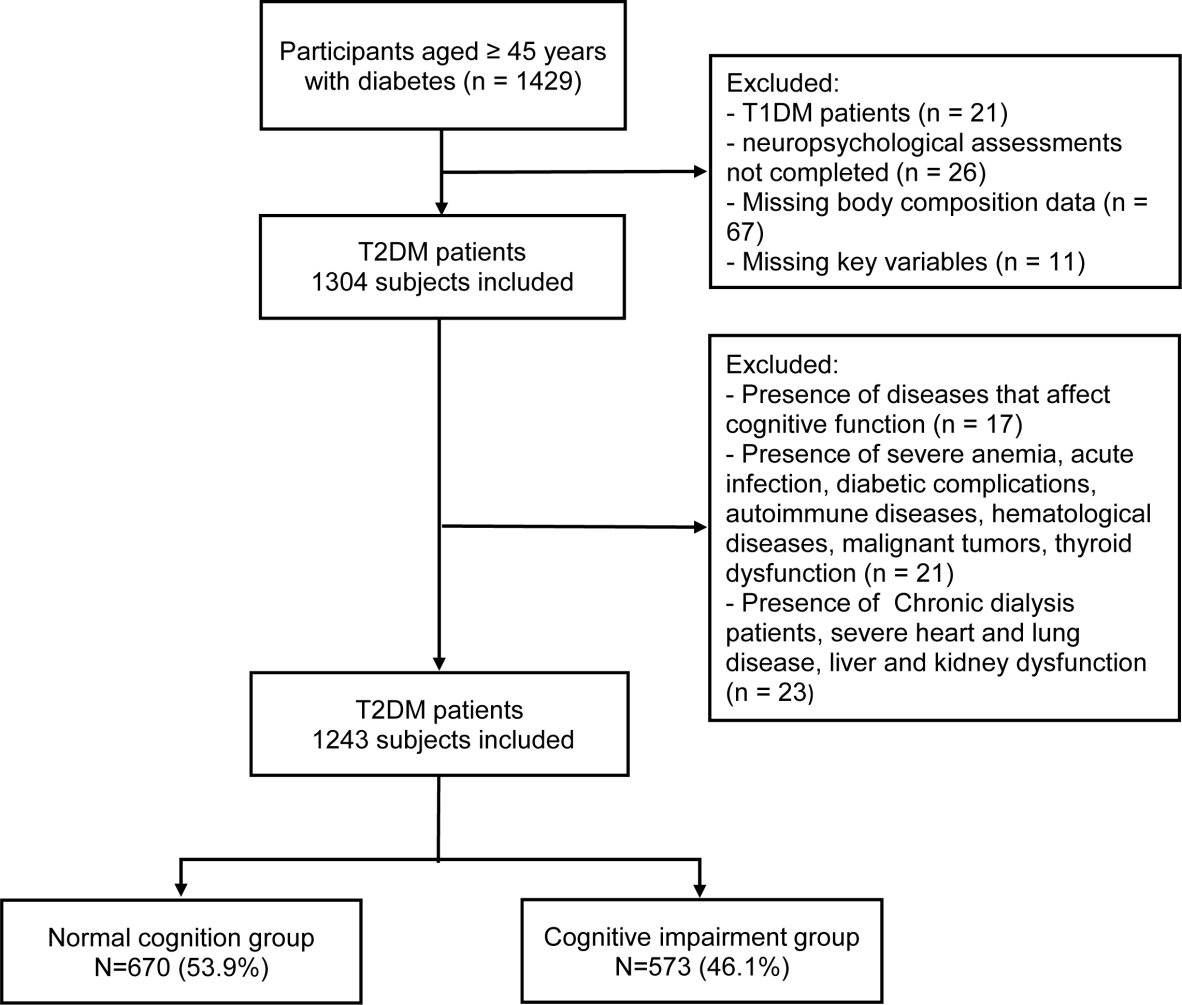
The flowchart depicting the selection of participants.

The following individuals were excluded from the study: Patients with Type 1 Diabetes Mellitus diagnosed at or below the age of 20 who were solely using insulin; patients unable to complete neuropsychological screening due to speech disorders, vision or hearing impairments, or unwillingness to cooperate; patients unable to undergo bioelectrical impedance analysis (BIA) due to the presence of metal objects in their bodies, edema, paralysis, or refusal to participate; patients missing key variables (BMI or WC); patients with conditions that could potentially affect cognitive function(head trauma, cerebral ischemia, severe depression, dementia, delirium, and schizophrenia); patients with severe anemia, acute infections, acute diabetic complications, autoimmune diseases, hematological disorders, malignancies, or thyroid dysfunction; and patients with an estimated glomerular filtration rate (eGFR) less than 30 mL/min/1.73 m², chronic dialysis patients, heart failure, severe pulmonary disease, or severe liver dysfunction. Ultimately, a total of 1243 patients were included. The detailed flowchart of the selection process is provided in **Figure 1**.

### 2.3 Assessment of CI

The Montreal Cognitive Assessment (MoCA) is a sensitive tool for early cognitive screening, demonstrating a specificity of up to 87% and sensitivity reaching 90% (35). A threshold score of 26 indicates cognitive impairment (CI), with scores below this suggesting CI (36). To adjust for educational attainment, participants with 12 years or less of education received an additional point on their total MoCA score (35). The MoCA was administered by trained personnel under standardized conditions to ensure consistency in the assessment process. Evaluations were conducted in a quiet environment to minimize distractions. Ultimately, a total of 1,243 participants were categorized into two groups: the normal CI group (N=670, MoCA score ≥26) and the CI group (N=573, MoCA score <26).

### 2.4 Assessment of anthropometric measurements and body composition analysis

Before the assessment, participants were instructed to empty their bladders and wear lightweight clothing while standing barefoot on a body composition analyzer (InBody770, Biospace, Korea), gripping the handles to facilitate measurement through electrodes placed on the soles of their feet. Height and weight were measured using an automatic height and weight measuring device (Seca287, Seca, Germany). WC was measured with a tape measure at the midpoint between the lowest rib edge and the iliac crest. Advanced direct segmental multi-frequency BIA technology was employed to evaluate several key body composition indicators, including VFA, PBF, FFM, ASM, and BMR. Additionally, BMR was calculated using BIA, with the formula validated through indirect calorimetry measurements (37). In order to offset the effects of height and weight on BMR (38), we divided BMR by the square of height (cm^2^) and BSA. The calculation formula of the relevant indicators is as follows: (39)


BSA=0.0061×height(cm)+0.0124×weight(kg)−0.0099

$$BMI=weight\;\left(kg\right)/height^{2}\;\left(m^{2}\right)$$

### 2.5 Covariates

This study collected demographic characteristics (such as gender, age, education level, and marital status), lifestyle factors (including smoking, alcohol consumption, duration of diabetes, regular exercise, and dietary control for diabetes), and medication usage from participants through standardized questionnaires, face-to-face interviews, and medical record reviews. A comprehensive evaluation of complications and comorbidities was conducted, which included assessing dyslipidemia, diabetic microvascular complications (DMC), peripheral artery atherosclerosis (PAA), coronary heart disease (CHD), and cerebrovascular diseases (CVD). All data were gathered by trained professional healthcare personnel. Standardized methods were used to measure blood cell counts, hemoglobin levels, serum biochemistry, and urinary parameters. Detailed methodologies for data collection, laboratory measurements, and variable definitions are provided in **Supplementary Materials 1**.

### 2.6 Statistical analysis

The Kolmogorov-Smirnov test and Q-Q plot assessed the normal distribution of continuous variables. Normally distributed variables are presented as means ± standard deviations or medians (interquartile range), compared using Student’s t-test, while non-normally distributed variables are expressed as medians with interquartile ranges and analyzed using the Wilcoxon rank-sum test. Categorical variables are shown as absolute counts and percentages, compared using chi-square or Fisher’s exact tests.

To evaluate the independent associations of BMR and relevant adjusted indices (BMR/Height², and BMR/BSA), body composition (VFA, PBF, FFM, and ASM), BMI, and WC with the risk of CI, we performed binary logistic regression analysis. For the purpose of ensuring good model fit while balancing complexity and explanatory power, the Akaike Information Criterion and the Bayesian Information Criterion were utilized as evaluation metrics. With the aim of assessing multicollinearity among the body composition variables, the variance inflation factor (VIF) was calculated and any variables with VIF values exceeding 10 were excluded to mitigate significant collinearity. Furthermore, the Hosmer-Lemeshow test was employed to evaluate the goodness-of-fit of the logistic regression model. To investigate the potential non-linear relationship between BMR and the risk of CI, as well as to observe trends across different levels of BMR, each indicator was divided into quartiles, using the first quartile as the reference group to calculate odds ratios (ORs) for CI with 95% confidence intervals. Based on baseline differences and known metabolic risk factors affecting CI in T2DM (2, 40–42), three progressive adjustment models were established: Model 1 was unadjusted; Model 2 adjusted for sex, age, education and marital status; and Model 3 further adjusted for drinking status, smoking status, diabetes duration, regular exercise, diabetic dietary control, dyslipidemia, Uric acid, DMC, PAA, CHD, CVD, eGFR, systolic blood pressure (SBP), diastolic blood pressure (DBP), hemoglobin A1c (HbA1C), white blood cell count (WBC), hemoglobin (Hb), urea nitrogen related to creatinine (UREA/CREA), gamma-glutamyl transferase (GGT), alanine aminotransferase (ALT), and aspartate aminotransferase (AST) and the use of statins and diabetes medications. To address the issue of multiple comparisons, we applied Bonferroni correction to the results from logistic regression models.

To further investigate the potential nonlinear relationships between BMR and adjusted indices (BMR/Height², and BMR/BSA), body composition (VFA, PBF, FFM, and ASM), BMI, and WC with the risk of CI, we utilized a restricted cubic spline (RCS) model with the rms package in R. This model, based on generalized linear models, employs smooth curves to fit the data. The knots’ number and position were determined by the Akaike Information Criterion and data distribution characteristics, ensuring an optimal balance between fit and complexity. The model accounts for all the aforementioned confounding factors.

To explore the predictive performance of BMR-related indices on body composition, BMI, and WC concerning CI, we constructed Receiver Operating Characteristic (ROC) curves and calculated the area under the curve (AUC). We conducted bootstrap validation on the ROC analysis using 1000 resamples to ensure robust evaluation of the model’s predictive performance. We first plotted ROC curves for BMR-related indices (BMR, BMR/Height² and BMR/BSA), determining the AUC for each index, where a larger AUC indicates stronger predictive capability. Additionally, we also assessed the predictive performance of BMR-related indices when combined with body composition (VFA, PBF, FFM and ASM) or obesity indices (BMI and WC). We included the combination of joint indicators in the model, plotting combined predictive ROC curves and calculating the AUC for these combinations to compare against the performance of using BMR-related indices alone. Additionally, the DeLong test was employed to assess the AUCs across the different models.

In order to explore whether BMR can explain the inconsistency between muscle strength reduction and central obesity on CI in the background of fat accumulation, we performed a mediation analysis. This analysis assessed the potential mediating role of BMR/BSA in the relationship between body composition (FFM, ASM) and WC on CI. Using binary logistic regression as the foundational model, we utilized the mediation package to conduct the mediation effect analysis, applying a bootstrap method with 1,000 repetitions to estimate the Average causal mediation effect and average direct effect, along with bias-corrected confidence intervals. Through the mediation analysis, we decomposed the overall impact of body composition (FFM, ASM) and WC on cognitive impairment into direct effects and mediated effects via BMR/BSA, while also calculating the mediation proportions and significance testing. Additionally, we established multivariable adjusted models: Model 1 was unadjusted, while Model 2 accounted for all previously mentioned confounding factors. To enhance the robustness of our results, we also calculated E-values to assess the sensitivity of our findings to unmeasured confounding.

All statistical analyses were conducted using SPSS software (V. 25.0; IBM, Armonk, NY, USA) and R Studio software (version 4.3.2). The significance level was set as two-tailed, with a p-value of less than 0.05 indicating statistically significant differences.

## 3 Results

### 3.1 Clinical baseline characteristics

**Table 1** presents the clinical baseline characteristics of 1,243 hospitalized patients aged 45 and older with T2DM, among whom 573 (46.1%) exhibited CI. In terms of demographic characteristics, the CI group was significantly older, had a lower marriage rate, and fewer years of education compared to the NC group (all P < 0.001). Additionally, regarding personal lifestyle and clinical features, the CI group was more likely to have poor dietary control of diabetes, a lower prevalence of PAA, and a higher prevalence of CVD (all P < 0.05). However, there were no significant differences in SBP, DBP, current smoking or drinking status, regular exercise, DMC, or insulin use (all P > 0.05). Laboratory tests indicated that liver enzymes (GGT, ALT), Hb, and eGFR were significantly lower in the CI group (all P < 0.05). Conversely, no significant differences were observed in glucose metabolism indicators (FBG and HbA1c), WBC, AST, uric acid, and UREA/CREA (all P > 0.05). In terms of anthropometric measurements and body composition analysis, the CI group exhibited higher WC and PBF, along with lower FFM and ASM (all P < 0.05). However, there were no significant differences in BMI and VFA. Regarding BMR-related indices, the CI group had significantly lower values for BMR, BMR/Height², and BMR/BSA (all P < 0.05).

**Table 1:** Characteristics of the study population by cognition status.

Characteristics	NC N=670 (53.9%)	CI N=573 (46.1%)	P Value
Age (years)	61.05 ± 7.17	63.67 ± 7.40	<0.001^*^
Sex-male	371(55.4%)	265(46.2%)	0.001^*^
Married-yes	629(93.9%)	477(83.2%)	<0.001^*^
Current smoking-yes	202(30.1%)	161(28.1%)	0.249
Current drinking-yes	184(27.5%)	137(23.9%)	0.318
Education (years)	11.20 ± 2.70	9.97 ± 2.90	<0.001^*^
Duration of diabetes (years)	6(1,12)	7(2,15)	0.074
Regular exercise	471(70.4%)	421(73.7%)	0.194
Diabetic dietary control	475(71.1%)	348(61.1%)	<0.001^*^
Dyslipidemia-yes	372(55.6%)	283(49.6%)	0.034^*^
DMC-yes	269(40.1%)	256(44.8%)	0.226
PAA-yes	388(57.9%)	333(58.2%)	0.001^*^
CHD-yes	220(32.9%)	219(35.4%)	0.016^*^
CVD-yes	142(21.2%)	205(35.8%)	<0.001^*^
Antidiabetic agents-yes	599(89.5%)	498(86.9%)	0.521
Metformin	311(46.7%)	254(44.6%)	0.470
Sulfonylurea	179(26.9%)	133(23.4%)	0.158
Thiazolidinedione	19(2.9%)	12(2.1%)	0.405
Alpha-glycosidase inhibitors	382(57.4%)	360(63.3%)	0.034^*^
SGLT - 2i	23(3.5%)	17(3.0%)	0.645
DPP- 4i	141(21.2%)	124(21.8%)	0.791
Insulin	286(42.7%)	253(44.2%)	0.603
Statins-yes	67(10.0%)	66(11.6%)	0.557
FPG (mmol/L)	8.67 ± 3.13	8.40 ± 2.97	0.131
HbA1C (%)	8.88 ± 2.04	8.85 ± 2.17	0.809
WBC (10^9^/L)	6.27 ± 1.65	6.29 ± 1.73	0.823
Hb (g/L)	136.11 ± 15.4	132.78 ± 15.5	<0.001^*^
Uric acid (μmol/L)	293.77 ± 82.43	296.31 ± 86.87	0.599
eGFR (mL/min/1.73 m²)	120.37 ± 35.28	115.84 ± 33.75	0.022^*^
UREA/CREA	23.35 ± 11.33	23.14 ± 7.65	0.701
GGT (U/L)	26(19,40)	25(18,36)	0.049^*^
ALT (U/L)	20(15,30)	18(13,27)	<0.001^*^
AST (U/L)	17(14,23)	18(14,23)	0.170
SBP (mmHg)	132.58 ± 13.92	133.09 ± 13.18	0.509
DBP (mmHg)	79.19 ± 8.44	79.31 ± 8.73	0.815
BMI (kg/m²)	26.07 ± 3.62	25.74 ± 3.46	0.104
WC (cm)	90.73 ± 9.95	92.01 ± 9.18	0.019^*^
VFA (cm^2^)	102.68 ± 30.39	103.92 ± 28.55	0.463
PBF (%)	30.47 ± 7.56	31.73 ± 7.52	0.004^*^
FFM (kg)	50.76 ± 8.89	48.34 ± 8.78	<0.001^*^
ASM (kg)	21.01 ± 4.42	19.86 ± 4.35	<0.001^*^
BMR (kcal/d)	1472.62 ± 193.78	1411.12 ± 190.76	<0.001^*^
BMR/Height^2^	0.053 ± 0.004	0.052 ± 0.004	<0.001^*^
BMR/BSA	770.17 ± 52.27	753.11 ± 53.16	<0.001^*^

Data are presented as means ± standard deviations or medians (interquartile ranges) for continuous data and numbers (%) for categorical data. NC, normal cognitive; CI, cognitive impairment; SBP, systolic blood pressure; DBP, diastolic blood pressure; DMC, diabetic microvascular complications (including diabetic nephropathy and/or diabetic retinopathy); CVD, cerebrovascular disease (including hemorrhagic and ischemic stroke); PAA, peripheral arterial atherosclerosis; CHD, coronary heart disease; FPG, fasting plasma glucose; HbA1c, hemoglobin A1c; WBC, white blood cell count; Hb, hemoglobin; eGFR, estimated glomerular filtration rate; UREA/CREA, urea nitrogen related to creatinine; GGT, gamma-glutamyl transferase; AST, aspartate aminotransferase; ALT, alanine aminotransferase; BMI, body mass index; WC, waist circumference; VFA, visceral fat area; PBF, percentage of body fat; FFM, fat free mass; ASM, appendicular skeletal muscle; BMR, basal metabolic rate; BSA, body surface area. ^*^ *P <*0.05.

### 3.2 Comparison of BMR-related indices, body composition, and obesity indices by quartiles

**Figure 2:**
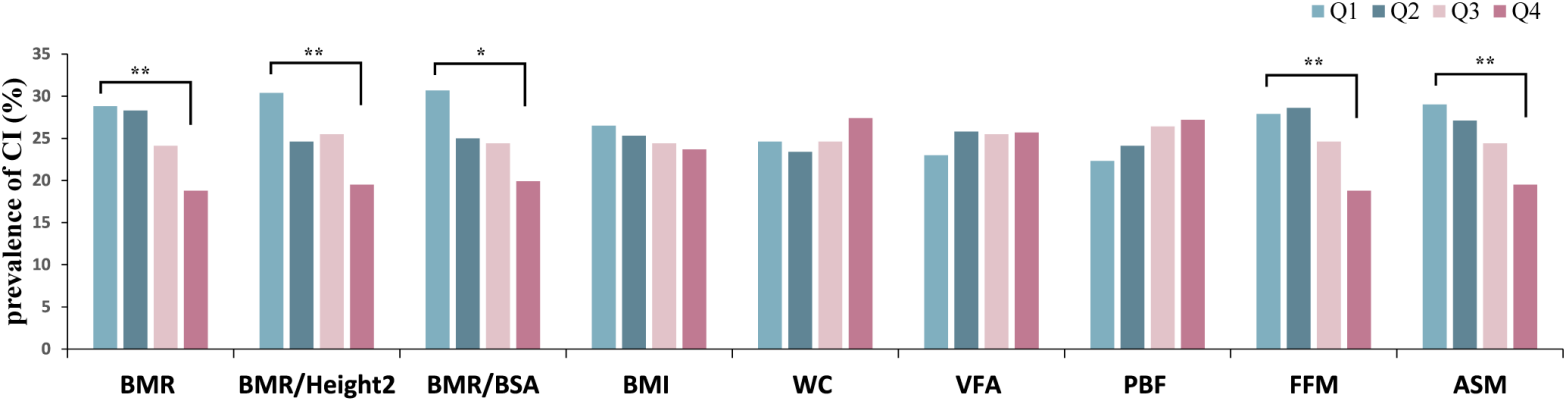
Prevalence of CI grouped by quartiles of body composition, obesity indices, BMR-related indices. CI, cognitive impairment; BMI, body mass index; WC, waist circumference; VFA, visceral fat area; PBF, percentage of body fat; FFM, fat free mass; ASM, appendicular skeletal muscle; BMR, basal metabolic rate; BSA, body surface area. Quartiles of BMR, Q1: 1008.42–1283.53, Q2:1283.54–1437.54, Q3: 1437.55–1583.96, Q4: 1583.97–1746.25; quartiles of BMR/Height², Q1: 0.041–0.049, Q2: 0.050–0.052, Q3: 0.053–0.055, Q4: 0.056–0.061; quartiles of BMR/BSA, Q1: 464.80–722.74, Q2: 722.75–761.17, Q3: 761.18–801.95, Q4: 801.96–864.71; quartiles of BMI, Q1: 17.58–23.53, Q2:23.54–25.54, Q3: 25.55–28.03, Q4: 28.04–42.52; quartiles of WC, Q1: 58.5–85.0, Q2: 85.1–90.0, Q3: 90.1–97.8, Q4: 97.9–130.0; quartiles of VFA, Q1: 34.67–82.65, Q2: 82.66–100.54, Q3: 100.55–119.97, Q4: 119.98–219.66 quartiles of PBF, Q1: 14.82–25.41, Q2: 25.42–30.99, Q3: 31.00–36.26, Q4: 36.27–51.53; quartiles of FFM, Q1: 31.3–42.2, Q2: 42.3–49.4, Q3: 49.5–56.2, Q4: 56.3–63.7; quartiles of ASM, Q1: 11.11–16.80, Q2: 16.81–20.37, Q3: 20.38–23.76, Q4: 23.77–27.16. ^*^ *P <*0.05, ^**^ *P <*0.001.

Participants were divided into four subgroups based on quartiles of BMR-related indices (BMR, BMR/Height², and BMR/BSA), body composition (VFA, PBF, FFM, and ASM), and obesity indices (BMI and WC). The prevalence of CI was then calculated for each of the four subgroups. The results indicated a decreasing trend in CI prevalence with increasing levels of BMR, BMR/Height², BMR/BSA, FFM, and ASM (as seen in **Figure 2**). Notably, participants in the fourth quartile for these metabolic indicators had the lowest prevalence of CI compared to those in the other three quartiles (all P < 0.05).

### 3.3 Independent associations of BMR-related indices, obesity indices, body composition, and CI risk

**Figure 3:**
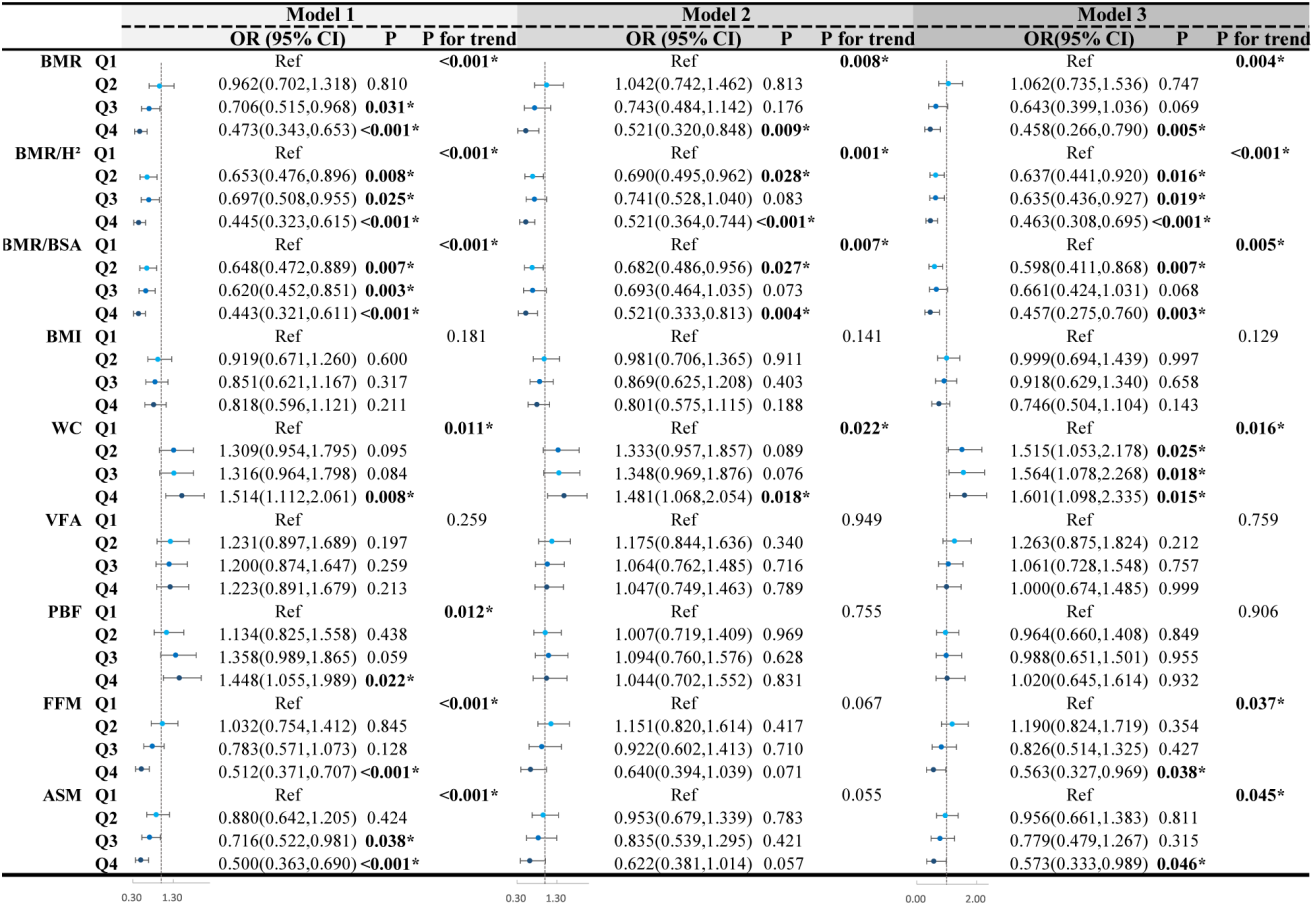
Logistic regression analyses of the association of quartiles of BMR-related indices, body composition and obesity indices with CI. OR, Odds ratio; 95% CI, 95% confidence interval; BMI, body mass index; WC, waist circumference; VFA, visceral fat area; PBF, percentage of body fat; FFM, fat free mass; ASM, appendicular skeletal muscle; BMR, basal metabolic rate; BSA, body surface area; BMR/H^2^, basal metabolic rate per height squared; CI, cognitive impairment. Model 1 was unadjusted. Model 2 was adjusted for sex, age, education and marital status. Model 3 was further adjusted for drinking status, smoking status, diabetes duration, regular exercise, diabetic dietary control, dyslipidemia, SBP, DBP, DMC, PAA, CHD, CVD, HbA1C, WBC, Hb, UREA/CREA, UA, eGFR, GGT, ALT, AST, use of statins and use of diabetes medications. ^*^ *P <*0.05.

This study employed multivariable-adjusted binary logistic regression analysis to explore the independent associations between BMR-related indices, body composition, obesity indices, and CI in middle-aged and older patients with T2DM. The results are presented in **Figure 3**. We categorized BMR and the adjusted related indices (BMR/Height² and BMR/BSA), body composition metrics (VFA, PBF, FFM, and ASM), and obesity indices (BMI and WC) into quartiles, using the first quartile as the reference group. In the unadjusted model, compared with the first quantile, BMR, BMR/Height², BMR/BSA, FFM, and ASM were significantly negatively correlated with CI risk when they were in the fourth quantile. Conversely, those in the fourth quartile for WC and PBF exhibited a significant positive correlation with CI risk (all P < 0.05). However, upon adjusting for demographic factors in Model 2, there was no significant association between body composition metrics (PBF, FFM, and ASM) and CI risk when positioned in the fourth quartile (all P > 0.05). Building on Model 2 by further adjusting for baseline differences and other metabolic risk factors, Model 3 retained the significant negative correlations between BMR, BMR/Height², BMR/BSA, FFM, and ASM with CI risk, alongside a significant positive correlation of WC with CI risk (all P < 0.05), generally demonstrating a clear linear trend in these associations. In Model 3, comparisons to the first quartile indicated that individuals with BMR (OR=0.458; 95% CI 0.266-0.790), BMR/Height² (OR=0.463; 95% CI 0.308-0.695), BMR/BSA (OR=0.457; 95% CI 0.275-0.760), FFM (OR=0.563; 95% CI 0.327-0.969), and ASM (OR=0.573; 95% CI 0.333-0.989) in the fourth quartile experienced a significant reduction in CI risk ranging from 42.7% to 54.3%. In contrast, individuals with WC in the fourth quartile faced a 1.6-fold increase in CI risk compared to those in the first quartile (OR=1.601; 95% CI 1.098-2.335). We performed Bonferroni correction to enhance the validity of the results (refer to **Supplementary Material 1**). The results indicated that in both Model 1 and Model 3, the fourth quartile of BMR, BMR/Height², and BMR/BSA was significantly associated with a lower risk of CI compared to the first quartile (all P < 0.05). The Hosmer-Lemeshow test results yielded a p-value of 0.567, indicating a good fit for the model.

### 3.4 Nonlinear associations among BMR-related indices, obesity indices, body composition, and CI risk

**Figure 4:**
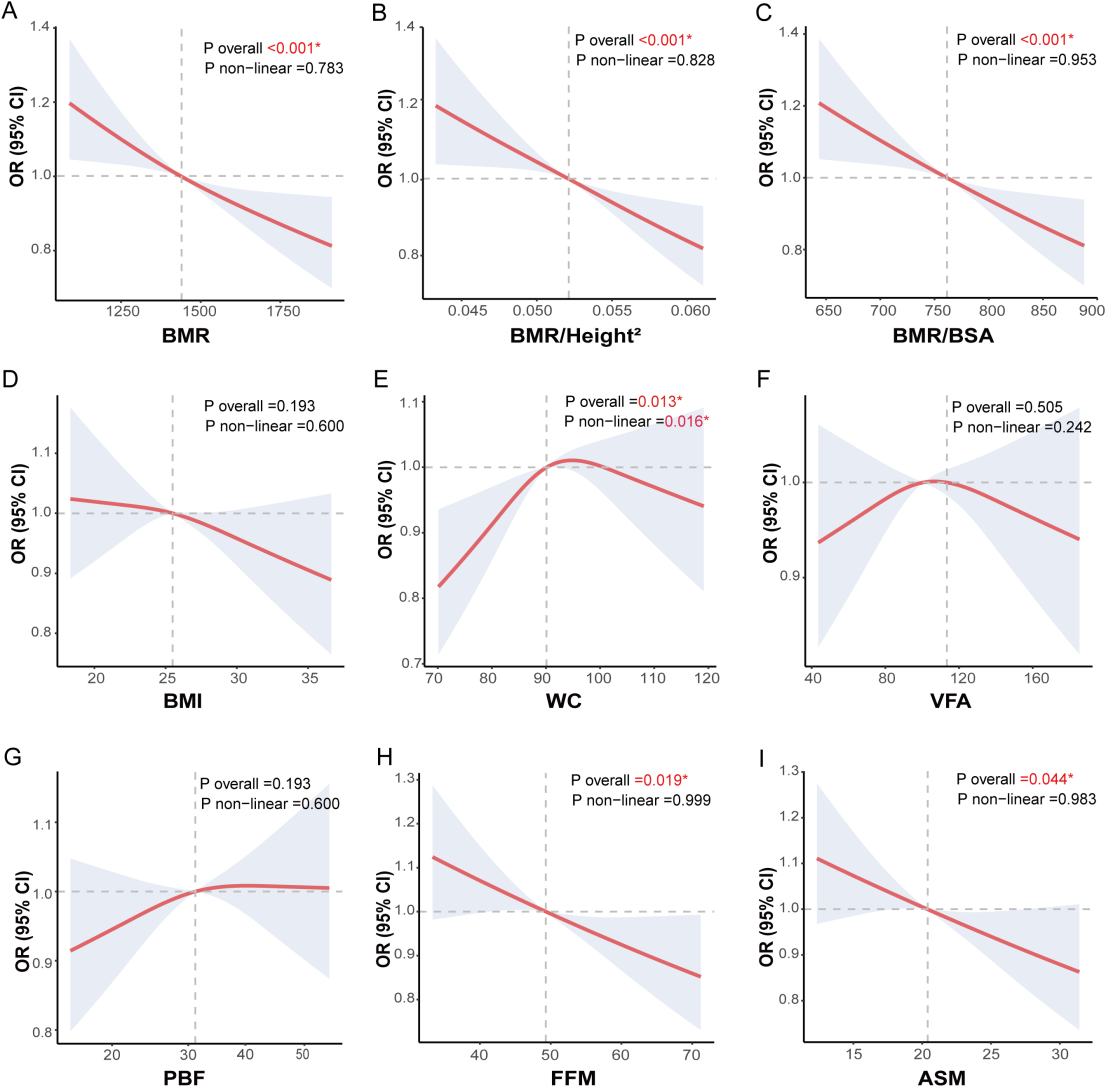
Nonlinear analysis of body composition, obesity indices, BMR-related indices and CI risk in middle-aged and elderly patients with T2DM. Nonlinear associations between body composition, obesity-related indices, basal metabolic rate-related indices and cognitive impairment (CI) in middle-aged and elderly T2DM patients were explored using restricted cubic spline (RCS) models and fitted with smooth curves. The three basal metabolic rate-related indices were **(A)** basal metabolic rate (BMR), **(B)** Ratio of basal metabolic rate to height squared (BMR/Height^2^), and **(C)** Ratio of basal metabolic rate to body surface area (BMR/BSA). The two obesity-related indices were **(D)** body mass index (BMI) and **(E)** waist circumference (WC). The four body composition indices were **(F)** visceral fat area (VFA), **(G)** percentage of body fat (PBF), **(H)** fat free mass (FFM) and **(I)** appendicular skeletal muscle (ASM). The models were adjusted for sex, age, education, marital status, drinking status, smoking status, diabetes duration, regular exercise, diabetic dietary control, dyslipidemia, SBP, DBP, DMC, PAA, CHD, CVD, HbA1C, WBC, Hb, UREA/CREA, UA, eGFR, GGT, ALT, AST, use of statins and use of diabetes medications.

This study utilized the RCS model to explore the potential non-linear associations between BMR and adjusted related indices (BMR/Height² and BMR/BSA), body composition metrics (VFA, PBF, FFM, and ASM), obesity indices (BMI and WC), and CI risk among middle-aged and older patients with T2DM. Adjustments were made for demographic factors, baseline differences, and other metabolic risk factors, with results presented in **Figure 4**. Consistent with the logistic regression findings, the dose-response relationship identified by the RCS model revealed significant negative correlations between BMR and the adjusted related indices (BMR/Height² and BMR/BSA) as well as body composition metrics (FFM and ASM) with CI risk. Specifically, as BMR, BMR/Height², BMR/BSA, FFM, and ASM increased, there was a significant linear decrease in CI risk, with no evident non-linear associations observed (all P overall < 0.05; all P nonlinear > 0.05). A slightly different result compared to logistic regression was that WC exhibited an inverted U-shaped curve association with CI risk (P overall = 0.013, P nonlinear = 0.016). Notably, we observed that when WC was ≤ 89.95 cm, the risk of CI increased with higher WC values; however, once WC exceeded 89.96 cm, the risk of CI began to decline as WC continued to rise.

### 3.5 Evaluation of predictive ability of BMR-related indices and their combination with obesity indices and body composition for CI risk

**Figure 5:**
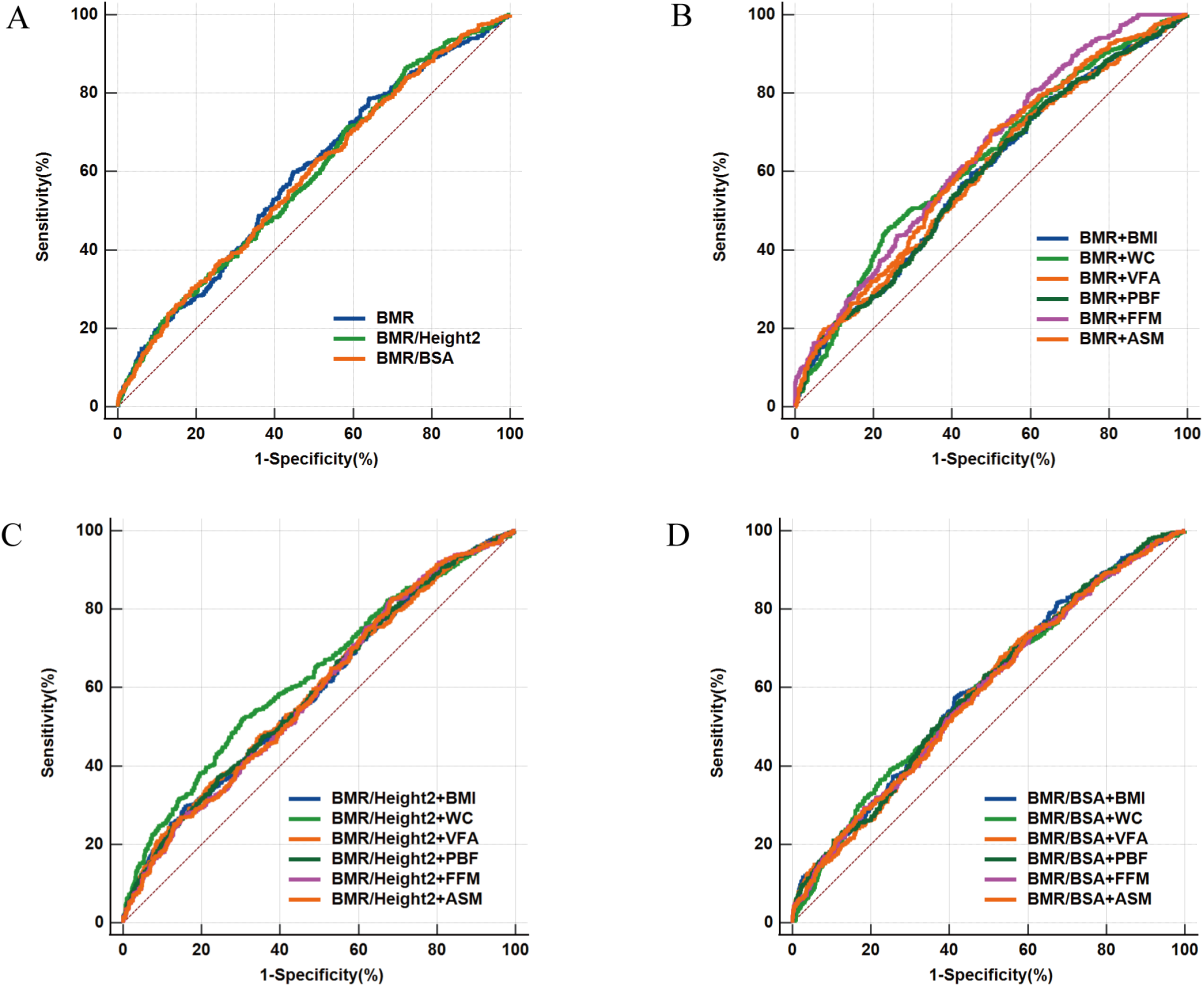
Predictive ability of BMR-related indices and their combination with obesity indices and body composition for CI in middle-aged and elderly patients with T2DM patients. **(A)** ROC curves illustrating the predictive ability of basal metabolic rate-related indices, including basal metabolic rate (BMR), Ratio of basal metabolic rate to height squared (BMR/Height^2^), and Ratio of basal metabolic rate to body surface area (BMR/BSA), for cognitive impairment (CI). **(B)** ROC curves showing the predictive ability of BMR in combination with obesity-related indices and body composition, including body mass index (BMI), waist circumference (WC), visceral fat area (VFA), percentage of body fat (PBF), fat free mass (FFM) and appendicular skeletal muscle (ASM), for CI. **(C)** ROC curves showing the predictive ability of BMR/Height^2^ in combination with obesity-related indices and body composition, **(D)** ROC curves showing the predictive ability of BMR/BSA in combination with obesity-related indices and body composition.

This study assessed three BMR-related indices, as well as their combined use with obesity indices and body composition to predict CI in middle-aged and elderly patients with T2DM. The results are presented in **Figure 5** and **Table 2**. Among all subjects, BMR, BMR/Height², and BMR/BSA, along with these indices in conjunction with BMI, WC, VFA, PBF, FFM, and ASM, significantly predicted CI in middle-aged and elderly T2DM patients (all P < 0.001). The AUC ranged from 0.585 to 0.645, with the highest AUC observed for the combination of BMR and FFM (AUC: 0.645; 95% CI: 0.615–0.677; P < 0.001). Using the maximum Youden’s index of 0.202, the cutoff value was determined to be 0.434. Utilizing the DeLong test to compare AUC values across different models revealed that the predictive ability for CI was significantly improved when combining BMR with WC, FFM, and ASM, compared to using a single BMR-related index. Specifically, the AUC range for the combination of BMR with WC was 0.627, while the AUC range for the combination with body composition indices (FFM and ASM) varied from 0.623 to 0.645. In contrast, the AUC range for individual BMR and adjusted related indices was only from 0.585 to 0.591.

**Table 2:** ROC analysis of BMR-related indices, body composition and obesity indices for predicting CI individually and in combination.

Indices	AUC (95% CI)	Cut-off	Sens. (%)	Spec. (%)	Youden Index	P value
BMR	0.591 (0.558,0.621)	1449.730	60.03 (55.9,64.1)	55.07 (51.2,58.9)	0.151	<0.001^*^
BMR/Height2	0.585 (0.552,0.615)	0.055	86.39 (83.3,89.1)	26.72 (23.4,30.2)	0.131	<0.001^*^
BMR/BSA	0.586 (0.552,0.616)	770.717	63.00 (58.9,67.0)	49.25 (45.4,53.1)	0.123	<0.001^*^
BMR+BMI	0.591 (0.560,0.624)	0.457	59.69 (55.5,63.7)	54.93 (51.1,58.7)	0.146	<0.001^*^
#BMR+WC	0.627 (0.597,0.660)	0.505	45.55 (41.4,49.7)	76.12 (72.7,79.3)	0.217	<0.001^*^
BMR+VFA	0.594 (0.563,0.627)	0.424	70.68 (66.8,74.4)	44.48 (40.7,48.3)	0.152	<0.001^*^
BMR+PBF	0.591 (0.559,0.624)	0.418	74.00 (70.2,77.5)	40.00 (36.3,43.8)	0.140	<0.001^*^
#BMR+FFM	0.645 (0.615,0.677)	0.434	79.58 (76.0,82.8)	40.60 (36.9,44.4)	0.202	<0.001^*^
#BMR+ASM	0.623 (0.591,0.655)	0.438	70.51 (66.6,74.2)	49.70 (45.8,53.6)	0.202	<0.001^*^
BMR/Height^2^+BMI	0.591 (0.559,0.623)	0.525	29.67 (26.0,33.6)	84.03 (81.0,86.7)	0.137	<0.001^*^
BMR/Height^2^+WC	0.630 (0.599,0.663)	0.492	52.01 (47.8,56.2)	69.55 (65.9,73.0)	0.216	<0.001^*^
BMR/Height^2^+VFA	0.593 (0.561,0.625)	0.513	35.43 (31.5,39.5)	77.76 (74.4,80.9)	0.132	<0.001^*^
BMR/Height^2^+PBF	0.593 (0.562,0.625)	0.509	37.17 (33.2,41.3)	75.67 (72.2,78.9)	0.128	<0.001^*^
BMR/Height^2^+FFM	0.587 (0.556,0.620)	0.407	82.55 (79.2,85.6)	31.64 (28.1,35.3)	0.142	<0.001^*^
BMR/Height^2^+ASM	0.587 (0.556,0.620)	0.409	82.02 (78.6,85.1)	31.94 (28.4,35.6)	0.140	<0.001^*^
BMR/BSA+BMI	0.599 (0.568,0.632)	0.466	57.42 (53.3,61.5)	58.51 (54.7,62.3)	0.159	<0.001^*^
BMR/BSA+WC	0.595 (0.565,0.628)	0.430	70.16 (66.2,73.9)	44.33 (40.5,8.2)	0.145	<0.001^*^
BMR/BSA+VFA	0.590 (0.559,0.623)	0.433	67.71 (63.7,71.5)	46.87 (43.0,50.7)	0.146	<0.001^*^
BMR/BSA+PBF	0.596 (0.564,0.629)	0.478	52.01 (47.8,56.2)	62.09 (58.3,65.8)	0.141	<0.001^*^
BMR/BSA+FFM	0.588 (0.556,0.621)	0.419	74.00 (70.2,77.5)	39.25 (35.5,43.1)	0.133	<0.001^*^
BMR/BSA+ASM	0.588 (0.555,0.620)	0.420	72.95 (69.1,76.5)	40.15 (36.4,44.0)	0.131	<0.001^*^

ROC, receiver operating characteristic; AUC, area under the ROC curve; 95% CI, 95% confidence interval; Sens, sensitivity; Spec, specificity; BMI, body mass index; WC, waist circumference; VFA, visceral fat area; PBF, percentage of body fat; FFM, fat free mass; ASM, appendicular skeletal muscle; BMR, basal metabolic rate; BSA, body surface area.

*Significance of AUC determined using Z-test, *P* < 0.05.

# Statistically significant differences were observed between the AUC of individual BMR-related indices (BMR/Height^2^, BMR/BSA, and BMR) and the AUC of combined BMR with obesity indices (WC, FFM, and ASM). (*P* values for the DeLong test were < 0.05).

### 3.6 Mediating role of BMR/BSA in the impact of WC, FFM, and ASM on CI risk

**Figure 6:**
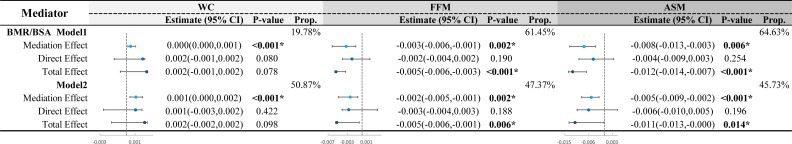
Mediation analysis of BMR/BSA in the impact of WC and body composition on CI in middle-aged and elderly patients. FFM, fat free mass; ASM, appendicular skeletal muscle; WC, waist circumference; BMR, basal metabolic rate; BSA, body surface area; CI, cognitive impairment. Mediation analysis was conducted using BMR/BSA as mediators. The total effect of body composition (FFM, ASM) and WC on CI was divided into direct effects and mediation effects through BMR/BSA. The mediation proportion and statistical significance were calculated. Model 1 was unadjusted. Model 2 was adjusted for sex, age, education, marital status, drinking status, smoking status, diabetes duration, regular exercise, diabetic dietary control, dyslipidemia, SBP, DBP, DMC, PAA, CHD, CVD, HbA1C, WBC, Hb, UREA/CREA, UA, eGFR, GGT, ALT, AST, use of statins and use of diabetes medications. In Model 1, the mediating effect E-value for WC is 2.39, for FFM is 2.85, and for ASM is 3.18. In Model 2, the mediating effect E-value for WC is 2.89, for FFM is 2.58, and for ASM is 3.03. ^*^ *P <*0.05.

This study investigates whether BMR/BSA can explain the inconsistent effects of reduced muscle mass and abdominal obesity on CI, particularly in the context of fat accumulation. As shown in **Figure 6**, we conducted a mediation analysis using BMR/BSA as the mediating factor to differentiate the total effects of WC, FFM, and ASM on CI into direct effects and the mediating effects of BMR/BSA. Regarding body composition metrics (FFM and ASM) affecting CI, we found that when BMR/BSA was included as a mediating factor, both models (Model 1 and Model 2) showed significant total effects of FFM and ASM on CI (with P-values < 0.05 for total effects). Additionally, there was a significant mediating effect of BMR/BSA (all P-values < 0.05), with the mediation proportion ranging from 45.73% to 47.37% after adjusting for covariates. Concerning the impact of WC on CI, when BMR/BSA was used as a mediating factor while adjusting for confounding factors, we found a significant mediating effect of BMR/BSA (P-value < 0.001), with a mediation proportion of 50.87%. However, no significant total effect was observed (P-value for total effect > 0.05).

## 4 Discussion

This study analyzed the associations between BMR-related indices (BMR, BMR/Height², and BMR/BSA), body composition (VFA, PBF, FFM, and ASM), obesity indicators (BMI and WC), and CI in middle-aged and older patients with T2DM using various models. Results revealed that CI patients had significantly lower levels of BMR, BMR/Height², BMR/BSA, ASM, and FFM compared to the NC group, while WC and PBF were higher. Multivariable-adjusted binary logistic regression and nonlinear analysis demonstrated a linear decrease in CI risk with increasing levels of BMR, BMR/Height², and BMR/BSA. Additionally, RCS curve analysis indicated a nonlinear association between WC and CI risk, presenting an inverted U-shaped curve with a turning point at 89.95 cm (P overall = 0.013, P nonlinear = 0.016). To explore whether BMR is a potential factor underlying the inconsistent effects of reduced muscle mass and central obesity on CI, we conducted ROC curve analysis and mediation analysis using combined indices. The ROC analysis demonstrated better predictive performance for CI when combining BMR-related indices with body composition or obesity indicators, with the joint analysis of BMR and FFM yielding the highest AUC of 0.645. These findings suggest that the coexistence of abnormal metabolism related to fat accumulation, reduced muscle mass, and altered BMR status may significantly increase the risk of CI. Furthermore, mediation analysis indicated that BMR/BSA plays a significant mediating role in the impact of WC, FFM, and ASM on CI risk, with mediation proportions ranging from 45.73% to 50.87%. This suggests that, in the context of fat accumulation, BMR may be a crucial explanatory factor for why reduced muscle mass and central obesity become significant risk factors for CI.

Previous studies have explored the relationship between BMR and neurodegenerative diseases, primarily focusing on the general population. For instance, a prospective study found low BMR as a significant risk factor for mild cognitive impairment (32). Additionally, a Mendelian randomization study indicated that lower BMR significantly increases the risk of Alzheimer’s disease (43). In contrast, our research extends these findings to middle-aged and older adults with T2DM. Our results are consistent with previous studies, indicating that lower BMR, BMR/Height², and BMR/BSA are all significantly associated with the risk of CI. This association may be attributed to insufficient energy supply leading to Aβ deposition, mitochondrial dysfunction, and impaired glucose metabolism, which in turn increases neuronal vulnerability and ultimately results in cognitive decline (44, 45). Therefore, our study further confirms the impact of low BMR on CI among middle-aged and older T2DM patients and emphasizes the importance of metabolic interventions targeting this high-risk population.

The relationship between WC as an indicator of central obesity and muscle mass reflected by ASM and FFM in relation to CI has been a topic of debate. Some studies suggest that both central obesity and decreased muscle mass consistently impact CI, significantly increasing the risk of CI. For instance, a meta-analysis involving 5,060,687 participants demonstrated that central obesity, characterized primarily by increased WC, significantly heightens the risk of CI (23). Several cross-sectional studies have also demonstrated that central obesity is a risk factor for CI (24, 46). Moreover, another meta-analysis found a significant association between decreased muscle mass and the risk of CI (47), while multiple cross-sectional studies further revealed a link between reduced muscle mass and cognitive decline (48, 49). However, some research suggests that neither central obesity nor muscle mass reduction is related to CI (25–27, 50). Our findings align with the former perspective: CI patients exhibited lower ASM and FFM alongside higher WC. Multivariable-adjusted RCS analyses indicated that increasing ASM and FFM levels correlated with a decreasing risk of CI among middle-aged and older T2DM adults. Nonetheless, previous studies have not thoroughly investigated the potential interactions between central obesity and muscle mass reduction. Our mediation analysis results indicate that BMR adjusted for BSA plays a significant mediating role in the relationship between WC, FFM, ASM, and CI risk, with mediation effects ranging from 45.73% to 50.87%. Related animal studies have shown a close link between fat accumulation and reduced energy expenditure (51, 52). Previous prospective and cross-sectional studies have highlighted that low BMR may increase the risk of cognitive decline, vascular dementia, and Alzheimer’s disease (32, 38, 53). Therefore, in middle-aged and older T2DM patients, BMR in the context of abnormal fat accumulation may be a critical factor contributing to the conflicting effects of central obesity and muscle mass reduction on CI.

This study found that BMR, BMR/Height², and BMR/BSA significantly predict CI in middle-aged and older patients with T2DM, with BMR showing the highest predictive effectiveness (AUC of 0.591). This aligns with research by Wang X et al., which also reported strong predictive capability of BMR for CI in individuals aged 65 to 85 years (32). Most current studies focus on the predictive abilities of individual BMR-related indices for various health outcomes. For example, Soysal P et al. demonstrated that BMR has good accuracy in predicting sarcopenia (38). Hsu W H et al. found that BMR provides stronger predictive value for osteoporosis in postmenopausal women (54). Similarly, Mao T Y et al. noted that BMR effectively predicts successful aging in older adults (55). Notably, there has been no research comparing the combined predictive efficacy of BMR with obesity indices or body composition in relation to CI risk. The ROC curve analysis in this study indicates that the combined predictive ability of BMR with body composition measures (FFM and ASM) or obesity indices (WC) significantly outperformed that of a single BMR- related index. Among these combinations, BMR combined with FFM demonstrated the highest predictive ability, with an AUC of 0.645. These results suggest that central obesity or low muscle mass, combined with low basal metabolic rate, may be significant risk factors for increased CI in middle-aged and older patients with T2DM. Although diabetic patients often exhibit insulin resistance and high resting energy expenditure (56), seemingly indicating a state of high energy metabolism, many patients actually experience an increase in body fat percentage alongside a decrease in muscle mass, ultimately leading to a reduction in overall BMR. This phenomenon is closely related to multiple factors, including the release of chronic low-grade inflammatory markers, excessive induction of reactive oxygen species, hepatic glucose production, abnormal fat distribution and metabolism, and long-term muscle wasting, which collectively reduce energy expenditure and significantly lower BMR (16–18). This study underscores the importance of using BMR-related indices in conjunction with obesity indices or body composition measures to predict CI risk in middle-aged and older T2DM patients. This finding emphasizes the need to consider BMR, muscle mass, and fat distribution comprehensively in diabetes management to effectively reduce the risk of CI and further improve patients’ overall health status.

## 5 Strengths and limitations

This study is the first to employ a mediation analysis model to systematically explore the comprehensive effects and interactions of BMR in relation to the risks of CI among middle-aged and older patients with T2DM due to declines in muscle strength and central obesity. Our findings provide new insights for developing more rational treatment strategies for CI. However, we acknowledge that our cross-sectional design imposes significant limitations on our ability to establish causal relationships, especially in the context of mediation analysis, which relies on the assumption of temporal precedence. Without establishing the temporal order of the variables, the validity of the mediation pathway is compromised, thereby increasing the likelihood of reverse causation. Moreover, limitations in the variables studied hinder a comprehensive understanding of the relationship between BMR and CI. Furthermore, the regional specificity of the sample may impact the generalizability of the results. To address these limitations, future research should adopt a prospective cohort design and focus on managing lifestyle factors and BMR for effective CI interventions in T2DM patients. Such longitudinal studies will be crucial for validating our mediation findings and establishing causal pathways.

## 6 Conclusion

The findings of this study indicate that low BMR is an independent risk factor for increased CI in middle-aged and older patients with T2DM. The combination of BMR and FFM is more effective in predicting CI. Furthermore, BMR/BSA serves as a significant mediating factor in the relationship between central obesity (WC), reduced muscle mass (ASM, and FFM), and the increased risk of CI. These results underscore the importance of integrated management of central obesity, muscle mass reduction, and BMR to effectively lower the risk of CI in patients with T2DM.

## Data Availability

The raw data supporting the conclusions of this article will be made available by the authors, without undue reservation.
