# Supplementary material for: The mediating role of body surface area-adjusted basal metabolic rate: effects of low muscle mass and central obesity on cognitive impairment in Chinese patients with type 2 diabetes mellitus
Source: Front Endocrinol (Lausanne). 2025 Jan 24;15:1513035. doi: 10.3389/fendo.2024.1513035 (PMC11802378; doi:10.3389/fendo.2024.1513035)
Supplement: Supplementary file 1 [file DataSheet1.docx]

**Supplementary Material 1**

Appendix to “The mediating role of body surface area-adjusted basal metabolic rate: Effects of low muscle mass and central obesity on cognitive impairment in Chinese patients with type 2 diabetes mellitus”

**Contents**

**Section 1:** **Data collection methods and variable definitions**-------------------------------------**3**

**Section 2:** **Bonferroni correction analysis**------------------------------------------------------------**3**

**Section 1:** **Laboratory measurement and data collection methods**

We collected the following demographic characteristics, lifestyle factors, and medication status of participants through standardized questionnaires, face-to-face interviews, and medical record reviews. Information on sex, age, education level (illiterate 0 years, primary school 2-6 years, junior high school 7-9 years, high school or above ≥12 years), and current marital status (married, unmarried, divorced) were obtained through face-to-face interviews. Participants self-reported their current smoking and drinking status (current or non-smoker/drinker), regular exercise habits (defined as engaging in at least 150 minutes of moderate-intensity aerobic exercise, or 75 minutes of vigorous-intensity aerobic exercise, or a combination of both, at least twice a week), and dietary patterns for diabetes (defined as prioritizing complex carbohydrates and high-fiber foods while limiting the intake of simple sugars, sodium, and saturated fats, maintaining an appropriate balance of nutrient proportions, and ensuring caloric balance) using standardized questionnaires. Medical record reviews provided information on the duration of diabetes, medication usage (hypoglycemic agents, lipid-lowering agents), dyslipidemia (defined according to the guidelines of the American Association of Clinical Endocrinologists [1] as LDL-C ≥ 160 mg/dL or HDL-C < 40 mg/dL, total cholesterol ≥ 240 mg/dL, triglycerides ≥ 200 mg/dL), diabetes-related microvascular complications (DMC), peripheral artery atherosclerosis (PAA), coronary heart disease (CHD), and cerebrovascular disease (CVD).

Diabetes-related microvascular complications include diabetic nephropathy and/or diabetic retinopathy. CVD encompasses both hemorrhagic and ischemic strokes. Hypertension is defined as a blood pressure of ≥140/90 mmHg and/or the use of antihypertensive medications. When measuring systolic blood pressure (SBP) and diastolic blood pressure (DBP), participants are required to rest for more than 10 minutes. Blood pressure measurements are taken using an automatic arm blood pressure monitor (AC-05C, Ling Qian, China), with the average of three readings taken at three-minute intervals.

After the participants fasted for more than 10 hours, we collected peripheral venous blood samples for laboratory testing. A hematology analyzer (Sysmex, XE-2100, Japan) was used to determine white blood cell count (WBC) and hemoglobin (Hb). Glycated hemoglobin (HbA1c) was measured using an automated glycoprotein analyzer (HA-8180, ARKRAY, Japan). The estimated glomerular filtration rate (eGFR) was assessed through creatinine clearance tests. A fully automated biochemical analyzer (TBA-120FR, Toshiba, Japan) was employed to measure fasting plasma glucose (FPG), uric acid (UA), total cholesterol (TC), triglycerides (TG), high-density lipoprotein cholesterol (HDL-C), low-density lipoprotein cholesterol (LDL-C), urea nitrogen/creatinine (UREA/CREA), alanine aminotransferase (ALT), aspartate aminotransferase (AST), and gamma-glutamyl transferase (GGT).

**Section 2: Bonferroni correction analysis**

| **Table S1** Logistic regression analyses of the association of quartiles of BMR-related indices, body composition and obesity indices with CI | | | | | | | | |
| --- | --- | --- | --- | --- | --- | --- | --- | --- |
|  | **Model 1** | | | **Model 2** | | | **Model 3** | |
|  | **OR (95% CI)** | **P** | **OR (95% CI)** | | **P** | **OR (95% CI)** | | **P** |
| **BMR Q1** | Ref |  | Ref | |  | Ref | |  |
| **Q2** | 0.962(0.702,1.318) | 1.000 | 1.042(0.742,1.462) | | 1.000 | 1.062 (0.735,1.536) | | 1.000 |
| **Q3** | 0.706(0.515,0.968) | 0.279 | 0.743(0.484,1.142) | | 1.000 | 0.643 (0.399,1.036) | | 0.621 |
| **Q4** | 0.473(0.343,0.653) | **<0.001*** | 0.521(0.320,0.848) | | 0.081 | 0.458 (0.266,0.790) | | **0.045*** |
| **BMR/Height² Q1** | Ref |  | Ref | |  | Ref | |  |
| **Q2** | 0.653(0.476,0.896) | 0.072 | 0.690(0.495,0.962) | | 0.252 | 0.637 (0.441,0.920) | | 0.144 |
| **Q3** | 0.697(0.508,0.955) | 0.225 | 0.741(0.528,1.040) | | 0.747 | 0.635 (0.436,0.927) | | 0.171 |
| **Q4** | 0.445(0.323,0.615) | **<0.001*** | 0.521(0.364,0.744) | | **0.003*** | 0.463 (0.308,0.695) | | **0.004*** |
| **BMR/BSA Q1** | Ref |  | Ref | |  | Ref | |  |
| **Q2** | 0.648(0.472,0.889) | 0.063 | 0.682(0.486,0.956) | | 0.243 | 0.598 (0.411,0.868) | | 0.063 |
| **Q3** | 0.620(0.452,0.851) | **0.027*** | 0.693(0.464,1.035) | | 0.657 | 0.661 (0.424,1.031) | | 0.612 |
| **Q4** | 0.443(0.321,0.611) | **<0.001*** | 0.521(0.333,0.813) | | **0.036*** | 0.457 (0.275,0.760) | | **0.027*** |
| **BMI Q1** | Ref |  | Ref | |  | Ref | |  |
| **Q2** | 0.919(0.671,1.260) | 1.000 | 0.981(0.706,1.365) | | 1.000 | 0.999 (0.694,1.439) | | 1.000 |
| **Q3** | 0.851(0.621,1.167) | 1.000 | 0.869(0.625,1.208) | | 1.000 | 0.918 (0.629,1.340) | | 1.000 |
| **Q4** | 0.818(0.596,1.121) | 1.000 | 0.801(0.575,1.115) | | 1.000 | 0.746 (0.504,1.104) | | 1.000 |
| **WC Q1** | Ref |  | Ref | |  | Ref | |  |
| **Q2** | 1.309(0.954,1.795) | 0.855 | 1.333(0.957,1.857) | | 0.801 | 1.515 (1.053,2.178) | | 0.225 |
| **Q3** | 1.316(0.964,1.798) | 0.756 | 1.348(0.969,1.876) | | 0.684 | 1.564 (1.078,2.268) | | 0.162 |
| **Q4** | 1.514(1.112,2.061) | 0.072 | 1.481(1.068,2.054) | | 0.162 | 1.601 (1.098,2.335) | | 0.135 |
| **VFA Q1** | Ref |  | Ref | |  | Ref | |  |
| **Q2** | 1.231(0.897,1.689) | 1.000 | 1.175(0.844,1.636) | | 1.000 | 1.263 (0.875,1.824) | | 1.000 |
| **Q3** | 1.200(0.874,1.647) | 1.000 | 1.064(0.762,1.485) | | 1.000 | 1.061 (0.728,1.548) | | 1.000 |
| **Q4** | 1.223(0.891,1.679) | 1.000 | 1.047(0.749,1.463) | | 1.000 | 1.000 (0.674,1.485) | | 1.000 |
| **PBF Q1** | Ref |  | Ref | |  | Ref | |  |
| **Q2** | 1.134(0.825,1.558) | 1.000 | 1.007(0.719,1.409) | | 1.000 | 0.964 (0.660,1.408) | | 1.000 |
| **Q3** | 1.358(0.989,1.865) | 0.531 | 1.094(0.760,1.576) | | 1.000 | 0.988 (0.651,1.501) | | 1.000 |
| **Q4** | 1.448(1.055,1.989) | 0.198 | 1.044(0.702,1.552) | | 1.000 | 1.020 (0.645,1.614) | | 1.000 |
| **FFM Q1** | Ref |  | Ref | |  | Ref | |  |
| **Q2** | 1.032(0.754,1.412) | 1.000 | 1.151(0.820,1.614) | | 1.000 | 1.190 (0.824,1.719) | | 1.000 |
| **Q3** | 0.783(0.571,1.073) | 1.000 | 0.922(0.602,1.413) | | 1.000 | 0.826 (0.514,1.325) | | 1.000 |
| **Q4** | 0.512(0.371,0.707) | **0.001*** | 0.640(0.394,1.039) | | 0.639 | 0.563 (0.327,0.969) | | 0.342 |
| **ASM Q1** | Ref |  | Ref | |  | Ref | |  |
| **Q2** | 0.880(0.642,1.205) | 1.000 | 0.953(0.679,1.339) | | 1.000 | 0.956 (0.661,1.383) | | 1.000 |
| **Q3** | 0.716(0.522,0.981) | **0.342** | 0.835(0.539,1.295) | | 1.000 | 0.779 (0.479,1.267) | | 1.000 |
| **Q4** | 0.500(0.363,0.690) | **<0.001*** | 0.622(0.381,1.014) | | 0.513 | 0.573 (0.333,0.989) | | 0.414 |
| OR, Odds ratio; 95% CI, 95% confidence interval; BMI, body mass index; WC, waist circumference; VFA, visceral fat area; PBF, percentage of body fat; FFM, fat free mass; ASM, appendicular skeletal muscle; BMR, basal metabolic rate; BSA, body surface area; CI, cognitive impairment. Model 1 was unadjusted. Model 2 was adjusted for sex, age, education and marital status. Model 3 was further adjusted for drinking status, smoking status, diabetes duration, regular exercise, diabetic dietary control, dyslipidemia, SBP, DBP, DMC, PAA, CHD, CVD, HbA1C, WBC, Hb, UREA/CREA, UA, eGFR, GGT, ALT, AST, use of statins and use of diabetes medications. ^*^ *P* <0.05. | | | | | | | | |

**References**

1. Jellinger, P. S., Smith, D. A., Mehta, A. E., Ganda, O., Handelsman, Y., Rodbard, H. W., . . . Goldberg, R. (2012). American Association of Clinical Endocrinologists' guidelines for management of dyslipidemia and prevention of atherosclerosis. *Endocrine practice, 18*, 1-78.
